# Emerging trend of increasing spring frost damage for beech at higher elevations in the Jura Mountains: evidence from tree‐ring data

**DOI:** 10.1111/nph.70471

**Published:** 2025-08-19

**Authors:** Yann Vitasse, Lynsay Spafford, Joanna Reim, Frederik Baumgarten, Elisabet Martínez‐Sancho

**Affiliations:** ^1^ Swiss Federal Institute for Forest, Snow and Landscape Research WSL 8903 Birmensdorf Switzerland; ^2^ Oeschger Centre for Climate Change Research University of Bern 3012 Bern Switzerland; ^3^ Faculty of Forestry and Environmental Sciences University of New Brunswick Fredericton NB E3B 5A3 Canada; ^4^ Department of Environmental Science‐Botany University of Basel 4056 Basel Switzerland; ^5^ Department of Biological Evolution, Ecology and Environmental Sciences Universitat de Barcelona 08028 Barcelona Spain

**Keywords:** climate change, dendrochronology, European beech, false spring, late spring frost, phenology, tree rings

## Abstract

- Late spring frost (LSF) severely impacts tree growth and forest productivity, with global warming potentially altering LSF risk due to asymmetric changes in vegetation onset and frost timing. However, reconstructing past frost regimes with climatic and phenological data remains challenging.
- Using phenological models, high‐resolution climate and tree‐ring data, we identified damaging LSF on European beech at two sites in the Swiss Jura mountains over nine decades. A novel tree‐ring indicator, comparing frost‐sensitive beech with evergreen Norway spruce, allowed us to isolate LSF impacts from background climate signals.
- While no significant long‐term trend in the safety margin between leaf‐out and LSF was detected since 1930, negative margins – that is, leaf‐out preceding last frost – were frequent at the high‐elevation site in the last two decades. Our tree‐ring approach identified six damaging LSF events since 1991, exceeding twice the long‐term return rate. No lag effects of LSF were found, suggesting beech can tolerate episodic frosts.
- These findings demonstrate the potential of tree rings as bioindicators of past LSF events, offering an alternative to climatic and phenological records, which contain uncertainties that hamper LSF reconstructions. The increasing frequency of damaging LSFs raises concern about future frost risks in mountainous areas under climate change.

## Introduction

Extreme climatic events such as late spring frosts (LSFs) and hot droughts play a crucial role in shaping forest ecosystems, influencing their structure, composition, and long‐term dynamics (Körner *et al*., 2016; Knutzen *et al*., 2025). Extreme events not only may have immediate impacts, such as defoliation, branch dieback, or mortality, but also trigger long‐term ecological responses by altering competition, species distribution, and forest resilience. In this context, it becomes urgent to deepen our understanding of how forests respond to extreme events for predicting future ecosystem shifts, as their frequency and intensity are expected to increase with climate change.

Among extreme events, the impact of damaging LSFs, also known as ‘false spring’ (Chamberlain *et al*., 2019), has been understudied in the forestry sector, although their impacts are considerable on gross primary production (Meyer *et al*., 2024), tree vitality (Baumgarten *et al*., 2023), and biotic interactions, such as with herbivores, seed predators, pollinators, and birds (Inouye, 2000; Marquis *et al*., 2019). In temperate regions, damaging LSFs are rare because the phenology of trees has evolved with local climate such that bud break occurs at a time when the probability of damaging frosts is minimal (Lenz *et al*., 2016). However, when damaging LSFs occur, they can cause massive defoliation or damage flowers and developing fruits, leading to diminished overall fitness and performance of the affected trees (Gu *et al*., 2008; Vitasse & Rebetez, 2018; Lamichhane, 2021). Because of partial or complete defoliation, the produced assimilates and nonstructural carbohydrates (NSC) are preferentially allocated to rebuild the second cohort of leaves, and subsequently, trees prioritize NSC replenishment at the expense of primary and secondary growth (Baumgarten *et al*., 2023). Therefore, LSF events are generally associated with a substantial reduction in secondary growth, resulting in very narrow annual tree rings (Dittmar *et al*., 2006; Vitasse *et al*., 2019). When they occur at a large scale, they can cause significant declines in gross primary production (Meyer *et al*., 2024). The effect of frost injury on tree growth is generally very strong in the concomitant LSF year, but tree‐ring analyses of mature temperate trees have revealed a strong resilience to these events; that is, the capacity of growth to return to predisturbance conditions (Príncipe *et al*., 2017; Vitasse *et al*., 2019; Tonelli *et al*., 2023). This high resilience might result from a physiological compensation effect. For instance, recent studies have shown that in European beech, after complete defoliation due to frost, leaves from the second cohort had higher Chl content and photosynthesis rates than leaves from the first cohort and that senescence was delayed (Zohner *et al*., 2019; Baumgarten *et al*., 2023; Luo *et al*., 2025).

Despite the significant impact of LSF on tree performance, there is no consensus on whether the overall risk of LSF will intensify or lessen in temperate regions under future climate scenarios. There is consensus, however, that the frequency of LSF events varies regionally and has changed with climate warming. In recent decades, LSF risk has globally increased in Europe and Asia, while in North America, where the risk was historically higher, it has decreased (Zohner *et al*., 2020; Lamichhane, 2021). Within Europe, trends diverge across regions depending upon their continentality and topography, but also species‐specific phenological sensitivity to temperature, with more sensitive species being more exposed to LSFs (Ma *et al*., 2019). For European beech, increasing risk of LSFs has been observed at its southern margin after 1990 with the acceleration of global warming (Sangüesa‐Barreda *et al*., 2021) as well as in the core of its distribution at elevations higher than 800 m (Vitasse *et al*., 2018a).

Typical approaches to identifying past damaging LSF in forest trees are based on climate data, phenological observations (and modeling), and species‐specific frost resistance thresholds, for example, temperature causing 50% of damage (e.g. Augspurger, 2013; Lenz *et al*., 2016; Zohner *et al*., 2020). However, even with accurate data, retrospectively confirming damage to buds or leaves remains highly challenging for several reasons. First, the temperature measured at a standard weather station can significantly differ from the temperature experienced by leaf tissue or buds, depending on irradiance, clouds, wind, and air humidity (Vitasse *et al*., 2021; Peaucelle *et al*., 2022; Kirchhof *et al.*, 2025). A clear night sky with very little wind and low air humidity is conducive to strong radiative cooling, resulting in lower temperatures in plant tissues by several degrees than that measured under standard conditions, that is, at a height of 2 m, under shelter and with sufficient ventilation. It is especially effective toward higher elevations, where heat loss during the night is more pronounced in the absence of clouds, due to the thinner atmosphere, which can lower plant tissue temperature by up to 5°C (Scherrer & Körner, 2010). Second, frost tolerance varies considerably during bud and leaf development, with the most sensitive stage occurring when leaves emerge from the buds (Lenz *et al*., 2013; Neuner *et al*., 2019). Accurate leaf‐out timing is therefore crucial for estimating frost risk. However, most of the phenological models have an average uncertainty exceeding 7 d (Basler, 2016; Spafford *et al*., 2025), which approximately corresponds to the time required for buds to develop from the swelling stage to fully unfolded leaves under mild conditions. Since frost tolerance can decrease by several degrees during this development process (Lenz *et al*., 2013), aligning climate and phenological data remain particularly challenging. Third, the intraannual variability of spring phenology among trees within the same population is generally high, ranging from 2 to 3 wk (Denéchère *et al*., 2021; Charlet de Sauvage *et al*., 2022; Delpierre *et al*., 2024). This variability is rarely accounted for in phenology modeling (but see Lin *et al*., 2024) and further complicates the assessment of potential damaging frosts, as substantial differences in cold hardiness among individuals are expected at a given time. Interestingly, depending on the timing of frost in relation to the phenological stage, the earliest individuals are not always the most exposed to LSF, as mature leaves are more resistant than emerging ones. Because of all these challenges, the risk of frost damages is often studied statistically through proxies, often using the amount of warmth accumulated (as a proxy for vegetation development) at the time of the last frost (Vitasse & Rebetez, 2018; Zohner *et al*., 2020) or using both meteorological and phenological observations (Augspurger, 2013; Ma *et al*., 2019). These methods allow providing an overall assessment of the risk of LSF and to detect changes in its frequency over time. Nevertheless, these approaches lack validation of actual plant damage to confirm the occurrence of these potential frost damages, increasing uncertainty in the detected trends.

Annually formed tree rings offer a valuable archive of past environmental conditions and have been extensively used to reconstruct past climate, including temperature and precipitation patterns, and extreme events (Büntgen *et al*., 2011; Harley *et al*., 2017; Cailleret *et al*., 2019). Tree‐ring width is indeed controlled by various abiotic and biotic factors (Fritts, 2012). The main abiotic factors regulating tree growth are water availability, nutrients, temperature, and soil characteristics. Biotic factors that reduce growth include pests, pathogens, and intra‐ and inter‐specific competition (Büntgen *et al*., 2009; Fernández‐de‐Uña *et al*., 2015) but other biotic factors such as mycorrhiza or fertilizers from animal activity can also promote tree growth (Anthony *et al*., 2022). Due to the integrative nature of tree‐ring records, damaging LSFs also leave distinctive signatures on tree‐ring series, providing an opportunity to identify and assess their occurrence and intensity over the years. These signatures can manifest as abrupt growth reductions and/or malformed rings due to changes in cell ultrastructure (Helama, 2023). However, damaging LSFs events also remain difficult to identify in tree‐ring chronologies, as their signatures can be confounded with other extreme events such as droughts, which also produce narrow rings (Büntgen *et al*., 2010; Cook *et al*., 2015; Vitasse *et al*., 2019). Yet, accurate detection of damaging LSFs would substantially help to better calibrate dynamic vegetation models for assessing forest productivity (Meyer *et al*., 2024).

Here, we investigated the frequency of LSFs near the upper elevational limit of beech in the Jura mountains since 1930 by developing a beech LSF tree‐ring indicator. We address the following questions:

1. To what extent have the leaf‐out date, last spring frost, and the risk of potential frost damage increased for beech near its upper elevational limit in the Jura Mountains based on phenology modeling and climate data?
2. Can we identify damaging LSFs applying a tree‐ring‐based approach; and, if so, have they increased over time? Do they correspond to those detected based on climate and phenology alone?
3. Do damaging LSFs affect the radial growth of beech trees in subsequent years?

## Materials and Methods

### Study area

**Fig. 1:**
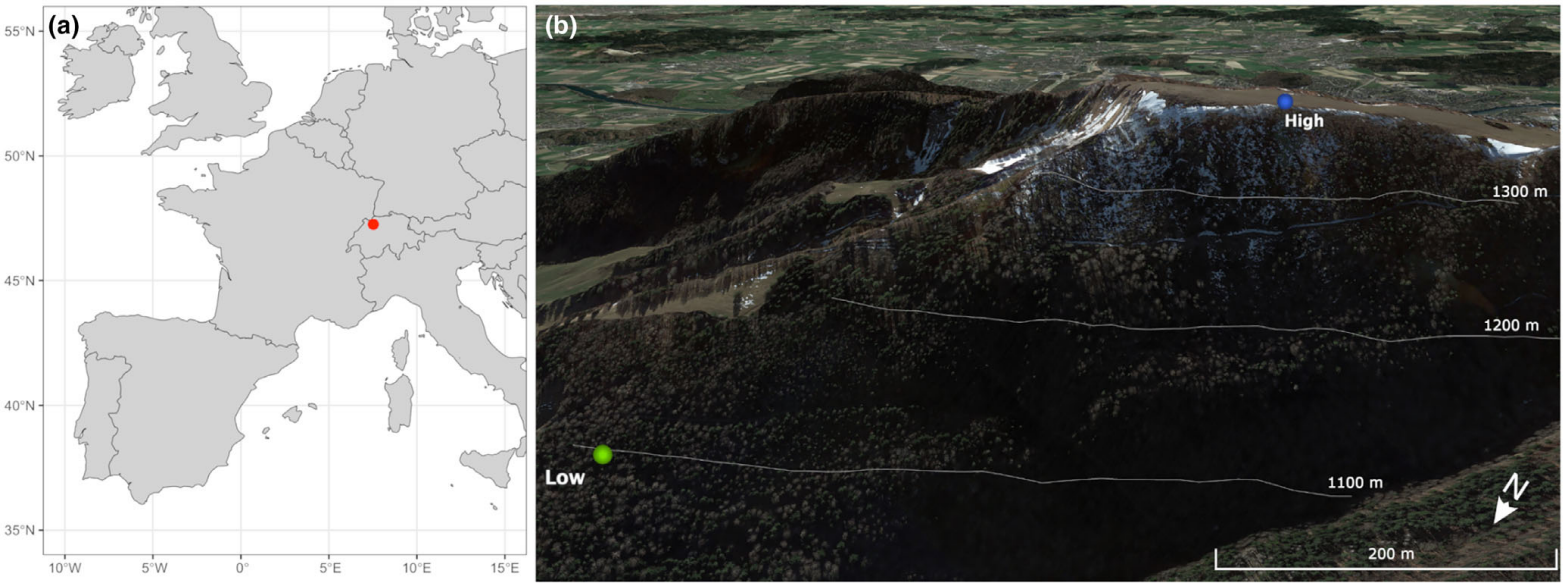
Location of the study sites in Europe (a) and in the Weissenstein mountain (b). 3D Map data: Google, Maxar Technologies.

Two sites located in the Jura Mountains in Switzerland were selected (Weissenstein area, 47°25′79″N, 7°52′64″E; 1085–1365 m above sea level (asl), Fig. 1), both dominated by European beech (*Fagus sylvatica* L.) and Norway spruce (*Picea abies* (L.) H. Karst). We specifically selected this area since on the 10^th^ and 11^th^ of May in 2020, a low‐pressure system over Europe brought cold air from the north, causing the temperature at this site to drop to a minimum of *c*. −2.0°C. As a consequence, beech trees lost all newly emerged leaves, with damages visible down to *c*. 1150 m asl (Supporting Information Fig. S1). The upper site is located at 1365 m, where beech foliage was severely affected in 2020 by the LSF, called hereafter the ‘high’ site, and the second site is on the same slope but at a lower elevation of 1085 m, where no freezing damages were observed in 2020, called hereafter the ‘low’ site. From 1931 to 1960, the mean July and January temperatures at the high site were 12.7°C and −4.2°C, respectively (low site: 14.6°C and −2.9°C), whereas from 1991 to 2020, they increased to 13.6°C and −1.8°C (low site: 15.6°C and −0.8°C). The mean annual precipitation at the high site was 1584 mm during 1931–1960 (low site: 1405 mm) and 1881 mm during 1991–2020 (low site: 1609 mm). Over the entire 1931–2020 period, the mean yearly temperature was 4.7°C at the high site and 6.3°C at the low site.

### Climatic data

We chose a gridded dataset for our analyses, established from the interpolation of Swiss weather station data recorded from 1930 to 2021 by the Federal Office for Meteorology and Climatology, MeteoSwiss. The interpolation algorithm from Thornton *et al*. (1997) was applied to these weather station data along with a 5‐m digital elevation model to account for the steep gradients in elevation which are present in the Jura mountains. The resulting 100‐m resolution gridded data product was provided by the Landscape Dynamics Lab at the Swiss Federal Institute for Forest, Snow, and Landscape Research. We extracted daily minimum, average, and maximum temperature as well as precipitation for our two sites along with sites above 800 m in Switzerland with available beech leaf‐out dates (see next sections) from this gridded dataset.

To assess how well our gridded dataset captured daily minimum temperatures, we compared the obtained values with those from a nearby weather station (47°15′N, 7°29′E) located at 1228 m asl; that is, 78 m above our low site and 137 m below our elevation site (period 1966–1973). There was a high correlation (Fig. S2, *R* ^2^ = 0.96) in daily minimum values of the gridded data and the measured weather station with consistent values based on elevation; that is, the temperature measured at the weather station was most of the time between the values of the low and high sites, with the high site being *c*. 1.15°C colder and the low 0.1°C warmer. Based on this validation, the gridded data were used for all subsequent analyses.

### Reconstruction of leaf‐out dates

We used leaf‐out dates of European beech observed from 1950 to 2022 from the long‐term volunteer phenology monitoring program managed by MeteoSwiss to train a phenology model for predicting leaf‐out dates at our two sites. At each site, one or several individual trees were observed one to three times on a weekly basis by Swiss Phenology Network volunteers (Bigler & Bugmann, 2018; Güsewell *et al*., 2018; Vitasse *et al*., 2018b). The leaf‐out dates were converted to day of year (DOY) values and averaged at the site level for a given year to produce a single record for each site‐year. Leaf‐out is defined as the date when 50% of the leaves are unfolded, corresponding approximately to the Biologische Bundesanstalt, Bundessortenamt und CHemische Industrie (BBCH) score 13 (Brügger & Vassella, 2003). We then selected all sites above 800 m elevation with at least 20 yr of observations, resulting in 42 unique sites and 2065 site‐years. We excluded lowlands in the calibration of the models because our two sites are situated at 1085 and 1365 m, and other limiting factors such as chilling and photoperiod may affect spring phenology at lower elevations, whereas we expect a major role of temperature at higher elevations for this species (Vitasse *et al*., 2018b).

We then used the R package phenor (Hufkens *et al*., 2018) to calibrate the M1 leaf emergence process model (Blümel & Chmielewski, 2012) on all 42 selected sites. This model has been found to perform best in the prediction of beech leaf out across Europe (Basler, 2016) and in Switzerland (Garnot *et al*., 2025; Spafford *et al*., 2025). The M1 model simulates leaf‐out as follows:

$$R_{\mathrm{frc}}\left(i\right)={\left(\frac{L_{i}}{10}\right)}^{k}\times \left\{\begin{array}{c} T_{i}>T_{b}:T_{i}-T_{b}\\ T_{i}\leq T_{b}:\;0 \end{array}\right.$$

$$S_{\mathrm{frc}}=\sum_{i=t_{0}}^{n}R_{\mathrm{frc}}$$

$$S_{\mathrm{frc}}\geq F_{\text{crit}}$$

where $t_{0}$ is the starting date for forcing accumulation, $T_{i}$ is the daily mean temperature, $T_{b}$ is the base temperature for accumulation, $L_{i}$ is the daylength, and *k* is a response parameter for the influence of photoperiod on forcing accumulation. Once the state of forcing accumulation, $S_{\mathrm{frc}}$, surpasses a critical threshold, $F_{\text{crit}}$, leaf‐out occurs. We used the default parameter ranges in phenor, with two exceptions to reasonably constrain parameter space: forcing was only allowed to occur following 1 January, and the base temperature for accumulation had to be > 0°C (Chuine *et al*., 2013; Delpierre *et al*., 2016; Chuine & Régnière, 2017). To calibrate the M1 model, we used the pr_fit_parameters() function in phenor that invokes the gensa optimization function within the gensa package in R (Xiang *et al*., 2013). Here we configured the general simulated annealing calibration to seek the global minimum root mean‐squared error (RMSE) between modeled and observed phenology. The annealing had a starting temperature of 10 000°C and a maximum of 40 000 model calls, following Hufkens *et al*. (2018). A global performance assessment including training and testing with all sites above 800 m elevation with at least 20 yr of observations (2065 site‐years, Fig. S3) resulted in an RMSE of 7.9 d (Fig. S4), which corresponded to 2.8 d of improvement in RMSE relative to a null model (RMSE of 10.7 d) assuming a fixed mean date based on the training set. A 10‐fold cross validation of the M1 model trained and validated more broadly over all sites with at least 20 yr of observations (5382 site‐years) revealed an RMSE of 7.7 d, which is consistent with other studies using the M1 model (Basler, 2016; Garnot *et al*., 2025; Spafford *et al*., 2025), and outperforming the equivalent null model for this exercise by 3.1 d. The resulting optimal parameters were a start date of 3 January, a base temperature of 0.29°C, a *k* parameter of 4.76, and an $F_{\text{crit}}$ of 1248 units. The predicted leaf‐out dates occurred on average on DOY 130 (min: 115–max: 143) and DOY 138 (119–155) for the low and high sites, respectively (Fig. S5).

### Assessing frost risk with climate and phenological data

We assessed the potential risk of LSF based on the leaf‐out model predictions and daily minimum temperature from the gridded dataset. This was estimated by considering whenever a frost ≤ −1.0°C occurred in the period from 5 d before to 14 d after the predicted leaf‐out date. Potentially damaging thresholds should be carefully selected, as the freezing tolerance of buds is very dynamic from bud swelling to leaf emergence. In addition, the interpolation necessary for gridded datasets may obscure some frost events. To account for this, we used an inclusive rather than exclusive threshold. We used −1.0°C because we observed a very strong damaging frost in the high site in May 2020 when the minimum temperature from our gridded dataset indicates only −2°C. We expect leaves to be vulnerable to frost below this threshold from *c*. 5 d before the expected leaf emergence date to +14 d afterward. Before budburst, buds are still relatively well protected and have significantly higher frost tolerance (Augspurger, 2009; Lenz *et al*., 2013). Extending the window earlier than 5 d before budburst led to the inclusion of additional potential LSF events based on climate data, but these LSF did not correspond to growth anomalies in the tree‐ring data, suggesting that they were not damaging. This supports our choice of a more conservative window, as an overly inclusive window before leaf emergence would lead to increased false positives. Conversely, after leaf emergence, freezing resistance increases only gradually with leaf maturation (Lenz *et al*., 2013), while the probability of frost events declines sharply. This justifies extending the postbudburst window up to 14 d. Selecting −1°C is potentially overly inclusive, as emerging leaves of trees are known to be damaged *c*. −3°C to −8°C depending on species (Sakai & Larcher, 1987; Vitasse *et al*., 2014; Neuner *et al*., 2019). However, we decided to select a higher temperature threshold because during nights with clear skies, air temperature from standard weather stations can be several degrees warmer than bud tissue due to strong radiative cooling of the buds compared to the sheltered weather station (Groot & Carlson, 1996; Vitasse *et al*., 2021; Kirchhof *et al.*, 2025). Finally, we selected −1°C and not 0°C to avoid identifying an excessive number of potential frost years.

We examined the presence of temporal trends for the last frost below −1°C and for the predicted leaf‐out dates. However, to detect if the risk of frost has changed over time, it is more meaningful to examine whether there is a trend in the difference between the last spring frost and the leaf‐out dates every year, also known as the temporal safety margin (Lenz *et al*., 2013, 2016; Vitasse *et al*., 2018a), as these two events can be decoupled. The safety margin was then calculated as the predicted leaf‐out date minus the last frost event below −1.0°C following Lenz *et al*. (2013). Trends in predicted leaf‐out dates, last spring frost ≤ −1°C, and the safety margin during the study period 1931–2021 were quantified using Theil–Sen's slopes and tested for significance with Mann–Kendall tests. This approach is commonly used in climate trend analysis due to its robustness to outliers and nonnormally distributed data, compared to ordinary least‐squares regression (Martinez *et al*., 2012; Klein *et al*., 2016).

### Tree‐ring analyses

In October 2021, 12 individual beech and spruce trees were sampled at each site. Two cores per tree were taken at breast height and perpendicular to the slope using an increment borer. Wood cores were air‐dried, and their surfaces were prepared with a core microtome (Gärtner & Nievergelt, 2010). High‐resolution images of the cores were taken using an image‐capturing system (Gärtner *et al*., 2024) with a digital camera (Canon EOS 5DSR; Canon Inc., Tokyo, Japan) equipped with a 100 mm macro lens at a resolution of 5950 dpi. Examples of tree‐ring series from four cores of the two species since 2000 are shown in Fig. S6. Tree‐ring widths were measured using the software CooRecorder 9.5 (Cybis Elektronik and Data AB, www.cybis.se). The individual tree‐ring series were visually examined with CooRecorder and statistically verified with the software Cofecha (Holmes, 1983). One beech tree from the low site was discarded due to crossdating problems. The age‐related trends from each individual tree‐ring width series were removed by fitting a cubic smoothing spline with a 50% frequency cutoff at 30 yr using the R package dplr (Bunn, 2008), transforming them into tree‐ring indices (tree‐ring width indices, RWI). This procedure aims at preserving the climatic‐related signals recorded in the tree‐ring series while discarding long‐term frequency related to age and disturbances. Then, the two tree‐ring indices series per tree were averaged to build individual tree series. At the site level, individual series were also averaged using a biweight robust mean to build species‐specific site chronologies. The quality of the chronologies was characterized by using classical dendrochronological descriptive statistics and parameters (Table S1).

To isolate the LSF events from the regular climate signals, we compared the tree‐ring series of a frost‐sensitive species (in our case beech) to a chronology of a less sensitive species (spruce). Less sensitive species to frosts are typically evergreen conifers which exhibit a late spring phenology, and which generally retain older needles after a damaging LSF (Gazol *et al*., 2019). Although LSFs can damage the newly produced needles of Norway spruce, its evergreen habit allows older, frost‐tolerant needles to continue photosynthesizing and carbon assimilation, thereby buffering the impact on annual growth (Neuner, 2014). By contrast, deciduous species such as European beech rely entirely on newly flushed leaves for carbon assimilation in spring (Baumgarten *et al*., 2023). Based on this assumption, we subtracted the spruce chronology from each individual detrended beech series (RWI) at each site and converted them into *z* scores in order to highlight uncommon patterns between the two species. Furthermore, we used individual tree‐ring series for beech to capture the potentially high phenological variability within the population, which can lead to differing degrees of frost damage. By contrast, we used the mean spruce chronology as an integrative reference to isolate the shared climatic signal with beech. Although such methodology is novel to detect frost years, it is widely used in dendrochronology to identify, for instance, insect outbreaks by comparing tree‐ring‐derived growth patterns of the host and nonhost species (Lynch, 2012; Guiterman *et al*., 2020; Camarero *et al*., 2022).

A mean chronology from these new individual timeseries was also built for each site (called ‘residual’ chronologies hereafter). To evaluate the climatic drivers of growth in both species and in the residual chronologies, Pearson correlations were computed for each site. These correlations were calculated between the three chronologies (beech, spruce, and residual) and daily climatic parameters for the current year, covering the full period from 1931 to 2020. This analysis was performed using the R package dendrotools (Jevšenak, 2020). To identify the potential pointer years due to frost effects on beech growth, individual residual series having a *z* score value below −1 and −1.5 for a given year were considered potentially affected by moderate and severe LSF, respectively. Finally, years showing > 60% of the trees with potential LSF damages (i.e. having *z* scores < −1) for a given site were considered potential LSF events.

**Fig. 2:**
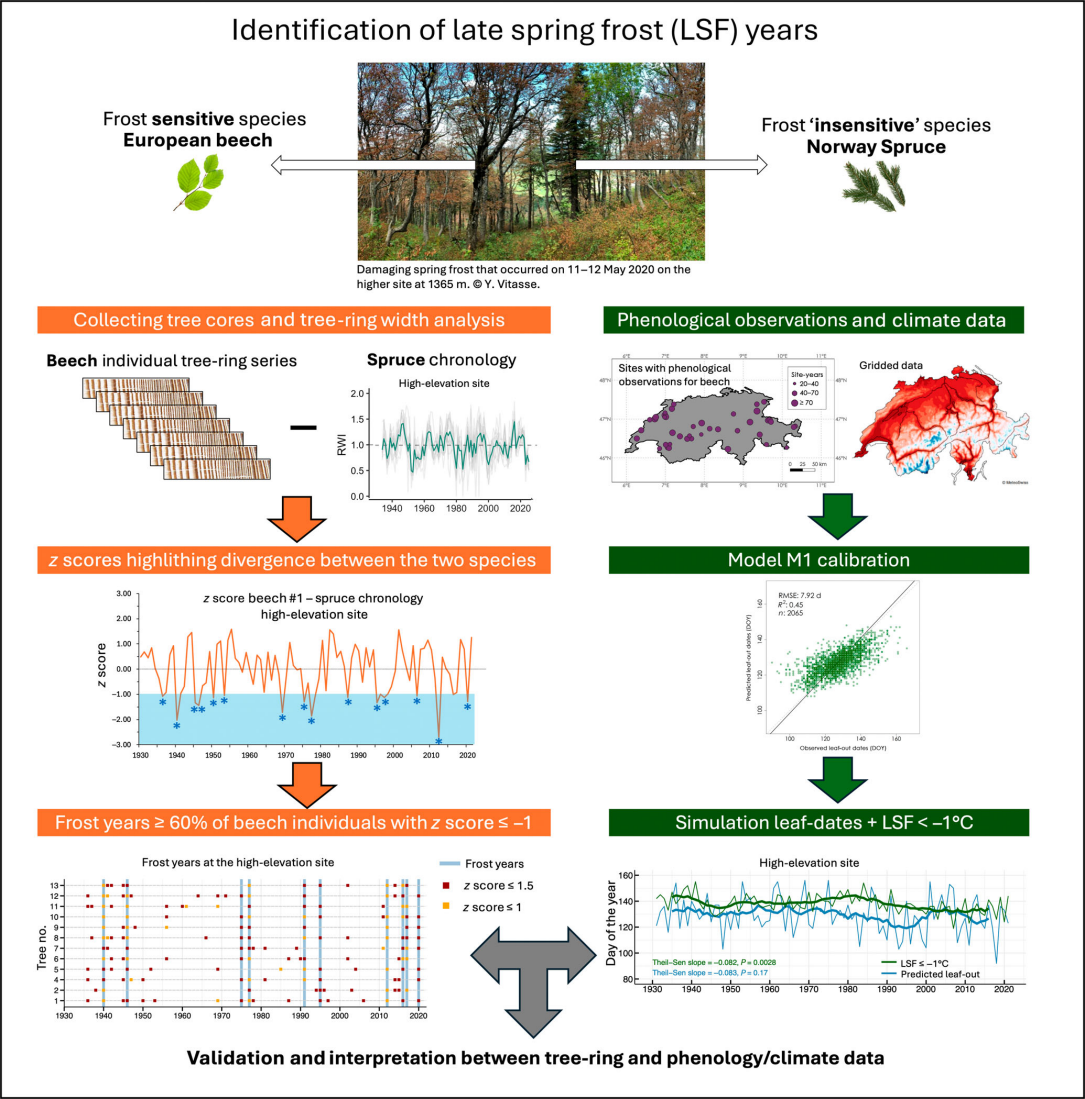
Conceptual overview of the methodological framework used in this study.

The legacy effects of LSF on the growth of beech and spruce were assessed using superposed epoch analysis (SEA) (Panofsky & Brier, 1958), by analyzing tree‐ring width deviations in the 4 yr following the identified LSF events of a given site. In SEA, tree‐ring parameters are expressed as scaled anomalies with respect to the mean RWI values. To determine if RWI for these years was significantly different from randomly selected sets of lags, bootstrap resampling was used to randomly select sets of lag years from the dataset and to estimate significances for the departures from the mean RWI. These analyses were carried out using the function *sea* from the dplr R package. Fig. 2 summarizes all the main different steps and analyses conducted in this study.

## Results

### Phenological‐based risk of late spring frosts

The occurrence of the last LSF below −1.0°C in spring has significantly advanced at the low site by −1.7 ± 1.1 d per decade (Theil–Sen slope ± 95% confidence interval) for the period 1931–2021, whereas no significant trend was detected at the high site (Fig. S5). This shift is particularly noticeable at the low site since the 1990s. For instance, the last LSF occurred after 24 April (DOY 115) in 75% of the years before 1990 but only in 31% of the years after 1990 for the low site (Fig. S5). By contrast, the predicted leaf‐out date of beech has significantly advanced over the entire analyzed period, by −0.70 ± 0.46 d per decade at the low site and −0.82 ± 0.56 d per decade at the high site (Fig. S5).

**Fig. 3:**
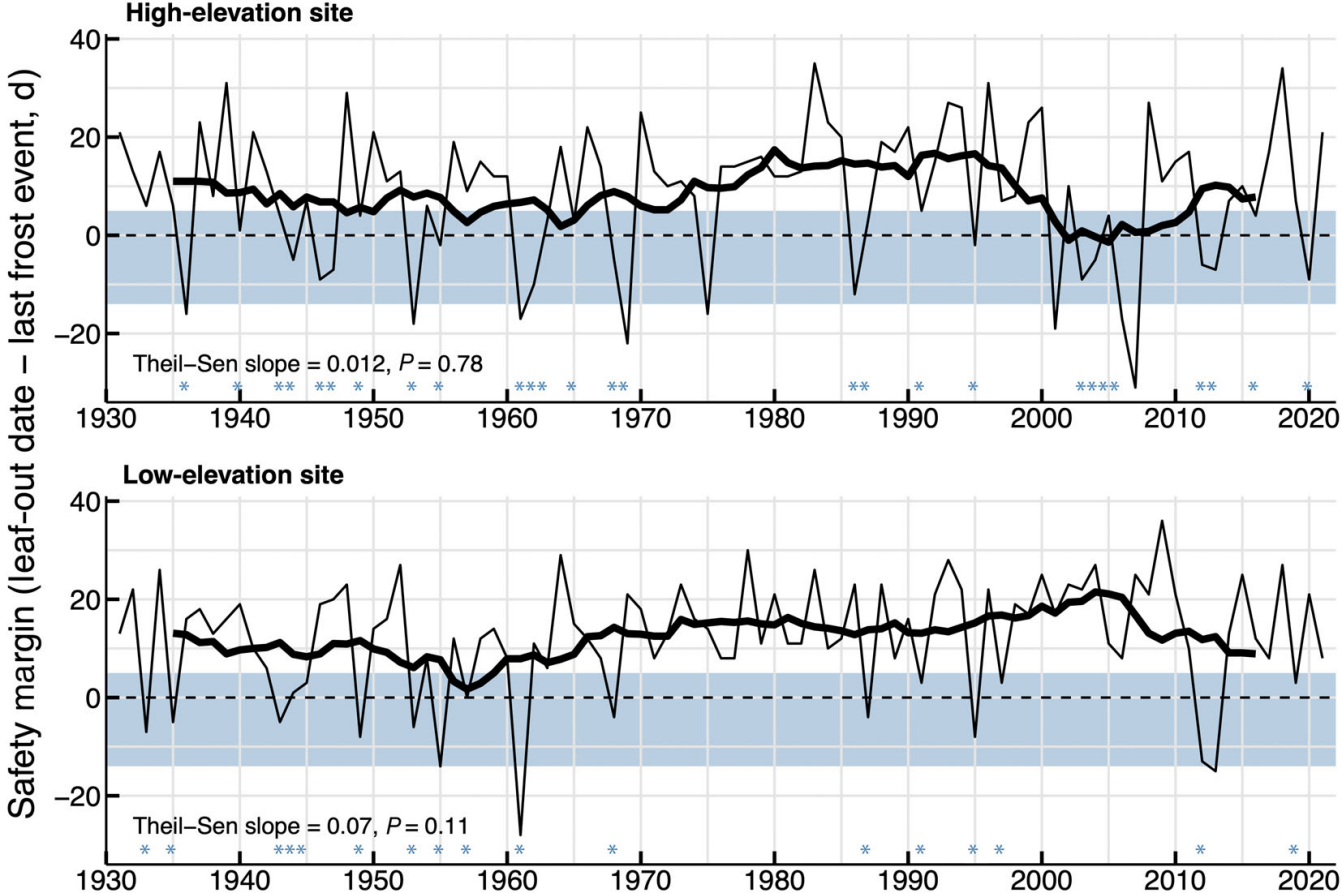
Safety margin against frost for the two elevation sites during the period 1931–2021. The safety margin was calculated as the predicted leaf‐out date minus the date of the last frost event below −1.0°C. Safety margin between −14 and +5 d around the date of leaf emergence, representing the window of vulnerability to frost, is shaded in steel blue. Ten‐year moving averages are represented with thicker lines. Blue stars indicate frosts ≤ −1.0°C occurring in the window of vulnerability to frost.

Overall, no significant trends were detected in the safety margin between the latest frost and the predicted leaf‐out dates over the study period at either site (Fig. 3). Potentially damaging LSFs (i.e. ≤ −1.0°C from 5 d before to 14 d after the predicted leaf‐out date) occurred on 17 (18.7%) and 27 (29.6%) out of the 91 yr analyzed at the low and high sites, respectively (Fig. 2). The average safety margin between leaf‐out dates and the last LSF < −1.0°C was +12.4 ± 1.2 d (mean ± SE) and +8.7 ± 1.4 d for the low and high sites, respectively. Interestingly, after a long period with a highly positive safety margin and few occurrences of potentially damaging LSFs (*c*. 1975–2000 for the high site and *c*. 1970–2010 for the low site), the safety margin shifts to values comprised between +5 and −14 d (i.e. potentially damaging frosts) on eight occasions during the period 2003–2021 at the high site but only on two occasions at the low site (Fig. 3).

### Tree‐ring climatic signals

**Fig. 4:**
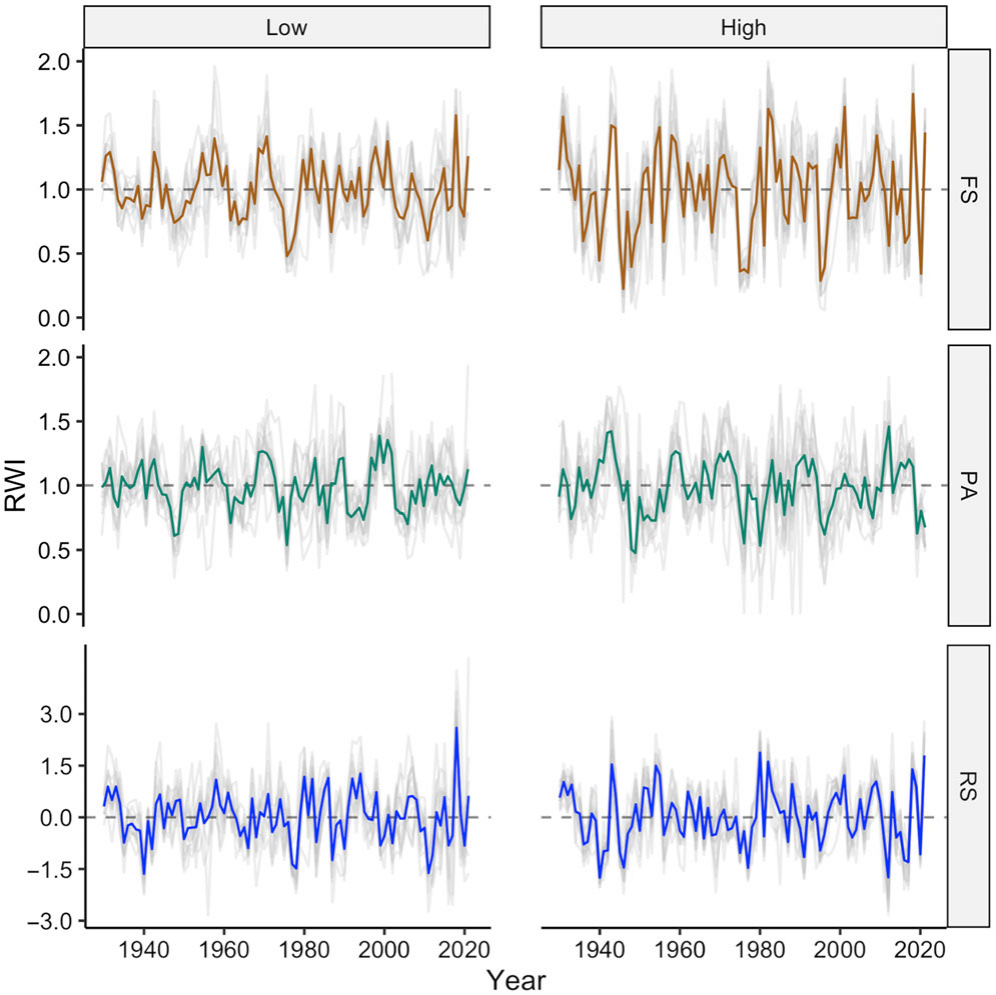
Individual tree‐ring width indices (RWI) of *Fagus sylvatica* (FS, high, *n* = 12; low, *n* = 11) and *Picea abies* (PA, high, *n* = 12; low, *n* = 12) as well as the individual residual series at the two study sites (gray lines) and the respective site chronologies (thicker lines). Residual series are calculated as the difference between each individual beech series and the spruce chronology of the same site converted to *z* scores.

Tree‐ring indices showed higher variability at the high site, especially for beech, with some years corresponding to extremely narrow rings, with an RWI below 0.40, such as in 1946, 1948, 1975, 1976, 1977, 1995, 1996, and 2020 (Fig. 4). Overall, as expected, beech and spruce showed similar growth patterns from year to year (high site *r* = 0.35 and low site *r* = 0.50, both with *P* < 0.001, Fig. S7), with some extreme years, however, diverging between the two species, especially at the high site. The residual chronologies (beech RWI – spruce chronology for each site) highlighted differences between the two species, showing values below −1 at the high site in the years 1940, 1945, 1946, 1975, 1977, 1991, 2012, 2016, 2017, and 2020.

**Fig. 5:**
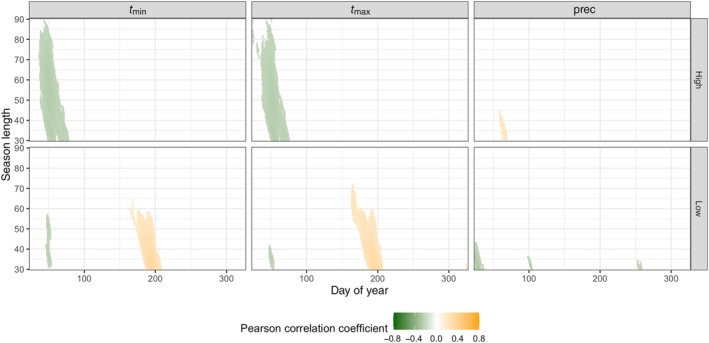
Pearson correlations between the site‐specific residual chronologies and daywise aggregated climate data (minimum (*t*_min_) and maximum (*t*_max_) temperatures and precipitation) in different window widths (season length) for the period 1930–2022. The residual chronology was calculated as the mean of all the individual beech ring width index series after subtracting the spruce chronology. The reference position for each value is the beginning of the considered window (season length). Only significant correlations (*P* < 0.05) are shown.

At the low site, the chronologies of the two species showed significant associations with precipitation and maximum temperature but differed in the season length and strength (Fig. S8). Spruce chronology showed significant negative correlations with maximum temperature and positive correlations with precipitation starting at DOY 160 and spanning from 30 to 90 d. For beech, these correlations were lower and also observed for the same days (DOY 160) but with different season length (*t*_max_ only from 30 to 40 d and precipitation from 30 to 70 d, Fig. S8). Beech chronology series also showed significant positive correlations with minimum temperature around DOY 200 for a window of 30–40 d. As a result, the residual chronology showed significant positive correlations with minimum and maximum temperature at DOY 200 for a 30–60‐d period (Fig. 5). This indicates that beech is less sensitive to heat and drought compared to spruce at low elevations.

At the high site, the growth of both species was barely affected by climatic parameters (Fig. S8). Only beech growth was significantly negatively associated with maximum temperatures that start DOY 50 and last for a period of 60–70 d, meaning a period including the temperatures of April and May. As a result, the residual chronology (obtained by subtracting the spruce chronology from each individual beech RWI series) at the high site showed negative correlations with warmer minimum and maximum temperatures before budburst (Fig. 5). In other words, under warmer conditions in late winter and early spring, spruce growth is more enhanced than beech growth at the high site, possibly as a consequence of damaging LSF events.

### Tree‐ring‐based identification of late spring frosts

**Fig. 6:**
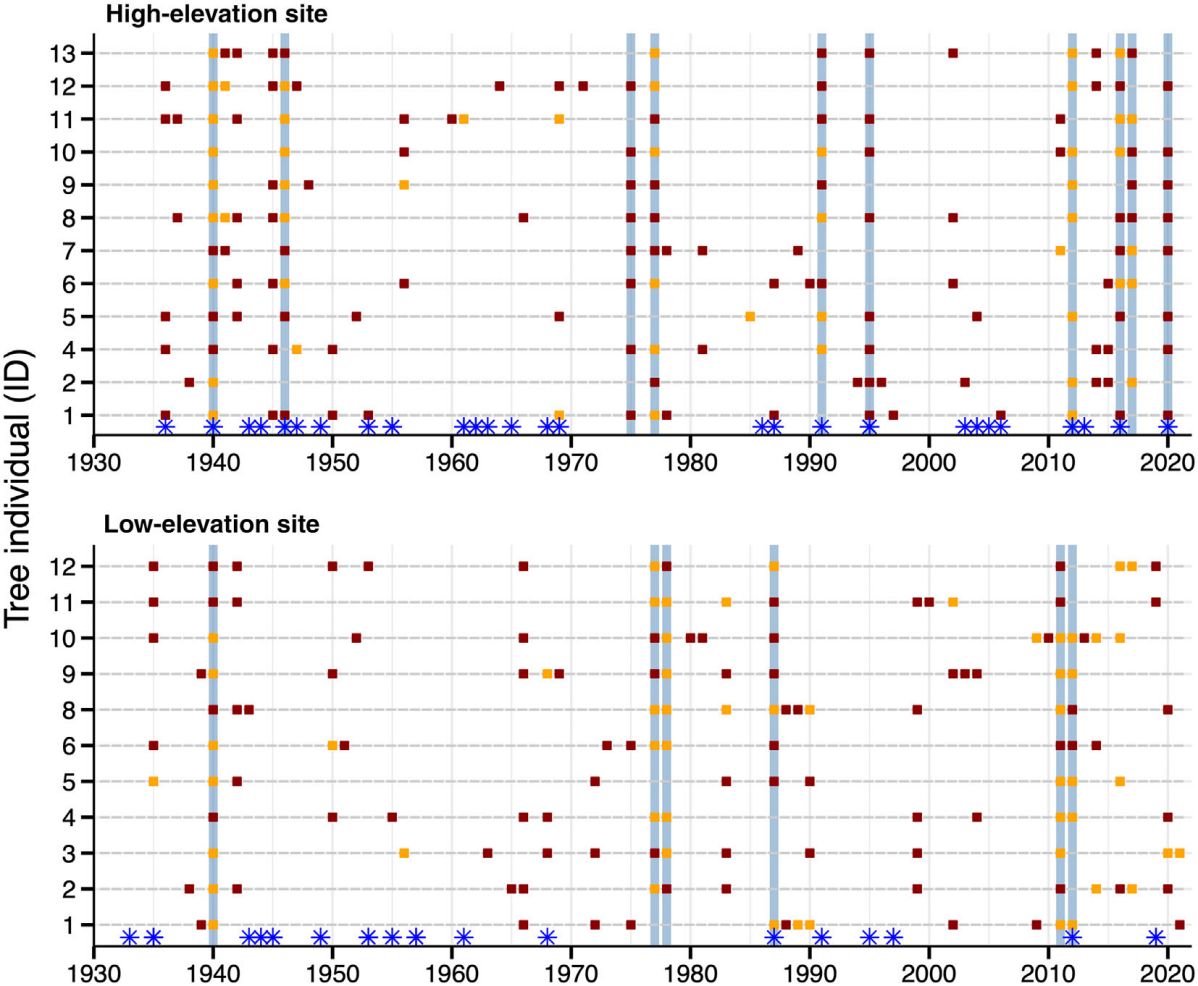
Identified damaging frost years based on the tree‐ring approach in the two studied sites since 1930. *z* Scores < −1.5, considered severe growth reduction, are represented in dark red, and *z* scores < −1.0, considered moderate growth reduction, are represented in orange. *z* Scores were calculated by subtracting the spruce chronology of the corresponding site from each individual beech ring width index series. Blue vertical lines represent years where at least 60% of the trees show *z* scores < −1. Blue stars correspond to potential frost events < −1°C occurring from −5 to +14 d from the predicted leaf‐out date.

After removing the common climatic signal between spruce and beech, we identified a potential frost year when *z* scores < −1.0 were observed for at least 60% of the cored beech trees. The years 1940, 1977, 1978, 1987, 2011, and 2012 were thus identified as potential frost years at the low sites, whereas 1940, 1946, 1975, 1977, 1991, 1995, 2012, 2016, 2017, and 2020 were identified for the high sites (Fig. 6). Note that in 2020, we observed complete defoliation due to an LSF that occurred in mid‐May at the highest site and no defoliation at the low site, which is consistent with the tree‐ring width and phenology/climate data (Figs 6, S5, S6). Seventy percent of the identified years by the tree‐ring approach were also identified as potentially risky based on the temperature and phenology data for the high site (i.e. frost ≤ −1°C occurring within −5 to +14 d from the simulated leaf‐out date), while only 2 out of the 6 yr identified from tree‐ring analyses aligned with the climate and phenological data for the low site (Fig. 6).

While only a few frost years were identified from the first 45 yr of the investigated period (1931–1976), that is, only one and three frost years at the low and high sites, respectively, we identified five (low site) and seven frost years (high site) during the following period from 1977 to 2021. Remarkably, four frost years were identified in the last decade from 2012 to 2021 at the high site, which might be the premise of a new trend for increasing risk due to a faster shift of phenology compared to the last frost.

### Do late spring frosts induce a legacy effect on radial growth?

**Fig. 7:**
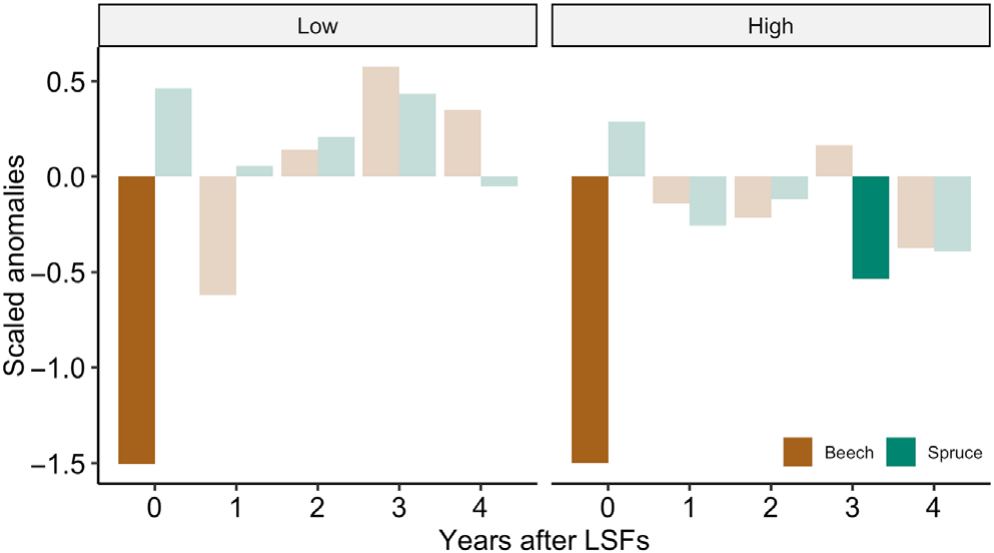
Superposed epoch analysis for species‐specific chronologies of beech and spruce at the high and low sites with corresponding identified late spring frost (LSF) events. Bars represent anomalies from the centered mean chronologies on the identified damaging LSF years. Dark bars indicate significant differences at *P* < 0.05 compared to randomly sampled nonevent years.

The SEA showed for both sites that a sharp decline in beech radial growth occurred in the years of identified LSF events while no significant negative anomalies were detected in the four following years (Fig. 7). By contrast, spruce growth did not show any significant reduction during the selected frost years (Fig. 7). Only at the high site was a significant growth reduction detected the third year following the identified LSF events.

## Discussion

Historically, damaging LSF events are rare but can cause massive damage to forest tree canopies, leading to reduced growth, flowering, and fruiting, and even mortality in saplings (Rodrigo, 2000; Dittmar *et al*., 2006; Baumgarten *et al*., 2023). In addition, building new leaves after LSF damage can take *c*. 2 months for beech (Tonelli *et al*., 2023) and typically leads to dramatic declines in sugar reserves (Baumgarten *et al*., 2023), which can limit the capacity of trees to defend against pathogens and insect pests as well as to withstand abiotic stress such as severe drought. As global warming is affecting both plant spring phenology and the occurrence of frost events, tracking damaging LSF over a long‐time period is relevant to detect any emerging trends and anticipate future changes. However, this task remains very challenging due to the complexity of determining these events based on phenological and climatic data and the scarcity of historical observations of frost damage to forest trees. Our methodology using tree‐ring series of spruce to leverage damaging LSF years on beech allowed us to clearly identify the LSF that occurred in 2020 at the high site, which caused complete defoliation (Fig. S1). Therefore, we believe that it offers a robust and valuable method for tracking such events over the long term. Our results show no major change in the occurrence of these damaging LSFs over the last nine decades, with a return rate of *c*. 20 yr at the low site and 10 yr at the high site, the latter potentially contributing to limiting the species to establish at higher elevations. Nevertheless, four damaging LSF events were detected at the high site in the last decade, and six have been recorded since 1991, exceeding twice the long‐term return rate. This trend raises concerns about an increasing risk in mountainous areas, driven by a systematically earlier phenology due to rising temperatures, combined with persistent, stochastic, and severe frosts at high elevations.

### The identification of late spring frost damage is challenging when using phenology and climate data only

Our study found a clear signal in tree growth induced by the LSF that occurred on 11–12 May 2020. Climatic data and phenological models have shown that the potential frost years identified by our tree‐ring analysis coincide with frost occurring around the date of leaf emergence in seven out of 10 cases for the high site, but only in two out of the six cases identified at the low site. However, many other years were also identified as potentially damaging from the climate and phenology data; that is, a frost < −1°C occurred around the dates when leaves were expected to emerge, while no reduction in growth was detected in the tree rings. This demonstrates that the identification of potential LSF using climatic and phenological data remains highly challenging due to mismatches between measured and actual plant tissue temperatures, uncertainties in phenology models, high individual variability in leaf‐out timing, and inaccuracy in gridded temperature data. Progress is needed in modeling plant tissue temperatures – such as bud meristems – by accounting for solar radiation, air humidity, or wind, as well as the physical characteristics of the tissue, including shape, size, and albedo (Peaucelle *et al*., 2022). Land surface temperature measured by satellites may offer a promising avenue to better capture the actual temperature experienced by vegetation, compared to gridded temperature data derived from interpolated weather stations. However, this approach also has limitations, including relatively low spatial and temporal resolution, difficulty in identifying the different species within a given pixel, and the relatively short time span of available data, which limits its applicability for assessing long‐term trends. Here, we used gridded temperature data in order to hindcast minimum daily temperature since 1930. Although this dataset has a very fine resolution and reproduces temperature accurately when comparing to the closest weather station (Fig. S2), it also contains some uncertainties and may slightly under‐ or overestimate frost events, making the use of temperature thresholds even more challenging.

We argue that our tree‐ring‐based approach provides a more robust method for detecting LSF damage by minimizing false positives. Indeed, when LSFs cause complete foliage loss, carbon gain halts temporarily, forcing trees to mobilize NSC reserves to rebuild their canopy. This recovery process, along with the need to replenish depleted NSC reserves, typically occurs at the expense of radial growth, resulting in narrow tree rings (Baumgarten *et al*., 2023). Beech was shown to be more negatively affected by LSFs than silver fir across elevational gradients (Cailleret & Hendrik, 2011; Gazol *et al*., 2019). Similarly, in Switzerland, sharp growth declines were observed in beech and oak when frost followed substantial heat accumulation in spring, while no such threshold was found for conifers including Norway spruce (Vitasse *et al*., 2019). Therefore, our approach highlights only years with strong growth anomalies between a sensitive and a nonsensitive species to frost damage, effectively ruling out common abiotic factors such as drought or floods. A closer examination of climatic data for years identified as frost years by our tree‐ring‐based approach but not by our climatological‐phenological method still reveals the presence of potentially damaging frost in most cases (Table 1). For instance, in 1977, a frost of −1.2°C occurred 8 d (6 May) before the simulated leaf‐out date at the low site. The high daily temperature amplitude (> 10°C) likely reflects clear‐sky night conditions, leading to strong radiative cooling (Table 1). Similarly, in 2011, two frost events with a daily temperature amplitude exceeding 10°C occurred on DOY 105–106, with minimum temperatures of −1.2°C and 1.6°C, respectively; that is, 10–11 d before the estimated leaf‐out date (Table 1). At the high site, only 3 yr (1975, 1977, and 2017) did not align with potential frost occurring within −5 to +14 d from the simulated leaf‐out date. Again, a closer examination of climate and phenological data suggests that these years may still have experienced damaging frost events. For instance, in 1975, on DOY 133, that is, 1 wk before the simulated leaf‐out date, a slight frost of −0.4°C was estimated in our gridded temperature data. The daily temperature amplitude exceeded 10°C, likely indicating cloudless conditions, which may have led to strong radiative cooling and lowered bud (or emerging leaves) temperatures below their freezing resistance (Table 1). Similarly, in 1977, *c*. 2 wk before the simulated leaf‐out date, temperature dropped to −2.8°C for several nights (Table 1).

**Table 1:** Potential damaging frost years as identified by tree‐rings analyses.

Years	*T*_min_ (°C, DOY, A)	Leaf‐out (DOY)	Likelihood of frost damage	Potential damaging frost outside of the selected window or with lower threshold (°C, DOY, A)
Low‐elevation site				
1940	−0.8, 138, A6.4	130	Likely	+0.0, 134, A12.8/−0.8, 138, A6.4 (−2.4°C at the high site)
1977	+0.8, 129, A6.0	134	Likely	−1.2, 126, A10.4
1978	+0.0, 133, A5.2	138	Likely	+0.0, 132, A8.8 (−2°C at the high site)
**1987**	**−1.6, 134, A8.8**	**130**	**Very likely**	
2011	+0.4, 124, A10.8	116	Likely	−1.2, 106, A12/−1.6, 105, A10.4/−3.2, 103, A9.6
**2012**	**−1.6, 138, A14.4**	**125**	**Very likely**	
High‐elevation site				
**1940**	**−2.4, 138, A6.4**	**141**	**Very likely**	
**1946**	**−2.0, 136, A10**	**128**	**Very likely**	
1975	−0.8, 152, A2.8	140	Likely	−3.6, 121, A12.8/−2.8, 125, A8/−0.4, 133, A10.4
1977	+1.2, 152, A12	143	Possibly	−2.8, 126, A9.2/−2.8, 128, A4.8/−1.6, 129, A5.6
**1991**	**−1.2, 144, A8.8**	**149**	**Very likely**	
**1995**	**−3.6, 135, A12.4**	**138**	**Very likely**	
**2012**	**−2.8, 138, A13.2**	**132**	**Very likely**	
**2016**	**−2.0, 136, A6.4**	**141**	**Very likely**	
2017	+0.4, 139, A9.6	136	Possibly	−8.4, 110, A8.8/−4.0, 117, A2/−3.6, 119, A8.8/−0.4, 129, A6.8
**2020**	**−2.0, 133, A9.6**	**124**	**Very likely**	

In bold are the years, where a frost < −1°C has been identified between −5 and +14 d from the simulated leaf‐out date. *T*_min_ corresponds to the minimum temperature found between −5 and +14 d from the predicted leaf‐out date. Leaf‐out corresponds to the predicted leaf‐out date with Model M1. Likelihood of frost damage correspond to our evaluation based on climate, predicted phenology, and tree‐ring analyses. The last column shows the potential frosts that may have caused the narrow tree ring. A, daily temperature amplitude °C.

### Are damaging late spring frosts increasing for European beech since 1930 and with the acceleration of global warming?

In many regions, phenology has advanced faster than the last spring frost due to accelerated global warming since the 1990s, increasing frost risk across the Northern Hemisphere (Zohner *et al*., 2020) including for beech in its southern range (Sangüesa‐Barreda *et al*., 2021) and at higher elevations in the Swiss Alps (Vitasse *et al*., 2018a). Our analysis of climate and phenological data does not indicate a significant increase in the frequency of potential damaging LSF events since 1930 at either site, as the safety margin has remained relatively stable over time. However, we found a lower safety margin at the high site, as also found in other studies (Bigler & Bugmann, 2018; Vitasse *et al*., 2018a), making beech more vulnerable to frost toward higher elevations. Notably, from 2001 to 2021, the safety margin has been negative (i.e. a frost < −1°C occurred after the leaf‐out date) on eight occasions, corresponding to an average return interval of *c*. 2.5 yr. This high frequency may show the premise of a trend toward increased risk of frost damage in the future. Consistently, the identification of damaging LSF events using our tree‐ring‐derived approach revealed six of these events after 1990, that is, every 5 yr on average, while it occurred only four times from 1930 to 1990, that is, every 15 yr. This is in line with previous studies, showing that the risk of spring frost is increasing for beech at higher elevations in Switzerland due to a stronger phenological shift than at lower elevation (Vitasse *et al*., 2018a,b), also reported at middle and high elevations in the Apennines in Italy or Spanish Pyrenees after 1990 (Bascietto *et al*., 2018; Allevato *et al*., 2019; Sangüesa‐Barreda *et al*., 2021; Tonelli *et al*., 2023). A higher frequency of frost damage often occurs at higher elevations due to the thinner atmosphere and reduced water vapor, which exacerbate radiative cooling during clear‐sky nights, causing bud or leaf tissues to drop below air temperature (Scherrer & Körner, 2010). This physical phenomenon highlights the need for careful interpretation of weather station data in frost risk assessments, particularly in mountainous or high‐altitude areas. Monitoring phenology and recording temperature data at these high‐elevation regions, where phenological shifts are generally stronger, and the risk of frost is higher due to stronger radiative cooling, is therefore particularly relevant in the context of ongoing climate change, and warrants further attention in future research. Remote sensing data and satellite observations hold promise for studying the frequency of damaging LSFs at these elevations and in other remote areas (Bascietto *et al*., 2018; Sangüesa‐Barreda *et al*., 2021). However, reconstructing past events remains challenging due to the limited spatial resolution of historical remote sensing products and the difficulty of aligning them with complex topography in steep mountainous terrain.

### Beech might be a particular species regarding frost risk

European beech is one of the most sensitive species to photoperiod and requires prolonged chilling exposure to fully break dormancy (Vitasse & Basler, 2012; Fu *et al*., 2013; Baumgarten *et al*., 2021). As a result, beech follows a more conservative phenology response to warming, exhibiting smaller shifts in leaf‐out dates compared to other co‐existing tree species (Fu *et al*., 2019), especially at lower elevations (Vitasse & Basler, 2012). This could be a safer strategy to avoid damaging LSF at lowlands. However, at higher altitudes, the long photoperiod prevailing at the time of leaf flush pushes the species to leaf‐out even when temperatures are not yet sufficient for other species (Vitasse *et al*., 2009), which may explain why frost damage to beech is often observed at higher altitudes compared to other species (Sangüesa‐Barreda *et al*., 2021). Our phenology M1 model captured this strategy by using a photoperiod parameter (*k*) which alters the accumulation rate of forcing required to budburst. Indeed, our calibrated process model demonstrated the relatively high importance of photoperiod on the timing of leaf‐out with a larger *k* value (4.76) than is typically observed for other species in Switzerland. For instance, using equivalent site selection criteria, the calibrated *k* values were 4.13, 3.03, 3.69, and 3.38 for common spruce, European larch, hazel, and horse chestnut, respectively (Spafford *et al*., 2025). Further, compared with a variety of commonly used process models, the M1 model with an inherent photoperiod constraint on forcing accumulation performed best for beech, consistent with its photoperiod sensitivity (Vitasse & Basler, 2012; Garnot *et al*., 2025; Spafford *et al*., 2025). However, deciduous species whose phenology is more sensitive to temperature than European beech may be more exposed to frost damage at all elevations and could be studied using a similar dendroecology approach. Our method should be applicable to other species, such as sessile or pedunculate oak, for which radial growth has also been shown to be significantly affected by LSFs in comparison to conifers (Vitasse *et al*., 2019).

### Limitation of the approach

The main assumption of our tree‐ring‐based approach is that European beech and Norway spruce share common climatic signals (Ježík *et al*., 2021), except for those related to LSF events. Our results validate that both species are sensitive to similar climatic cues, which is particularly true at the low site, whereas they only diverge in the ‘frost’ signals at the high site. At this elevation, warmer conditions in early spring may lead to an increasing risk of damaging frost due to premature bud burst. The higher sensitivity of spruce to temperature and precipitation during early summer compared to beech, especially at lower elevations, was also observed in another elevation gradient in central Europe (Sedmáková *et al*., 2019). By using the spruce chronology to identify damaging frost years for beech growth, we can distinguish frost‐related narrow rings from those caused by summer drought (common in both species). This was also found for silver fir compared to beech (Gazol *et al*., 2019). The fact that spruce is more sensitive to drought than beech makes our approach even more conservative and reliable, avoiding false positive frost years. However, whenever a damaging frost would occur in the same year as a severe drought, we may wrongly exclude it. Although the probability that both extreme events occur within the same year is extremely low, it will inevitably increase in the future as extreme droughts are expected to increase (Chiang *et al*., 2021), while the risk of frost is not decreasing (Zohner *et al*., 2020). The impact of this combination of stresses on forest integrity remains largely uncertain, but it could trigger an abrupt decline in growth and vitality, ultimately leading to tree mortality (Vanoni *et al*., 2016; Luo *et al*., 2025).

Another limitation of our study is the influence of masting, which can strongly affect radial growth in both spruce and beech (Drobyshev *et al*., 2010; Hacket‐Pain *et al*., 2019). In years when only beech experiences masting, it may cause false positives – apparent growth reductions compared to spruce unrelated to frost damage. In such cases, climate and phenological data are essential to confirm or rule out actual frost events. Nevertheless, the frost years we identified mostly coincide with LSFs occurring around the predicted leaf‐out date. This alignment suggests that masting had minimal influence on our results, likely because we applied a stringent growth reduction threshold that masting alone may not induce.

### Conclusions

By examining the historical trajectory of damaging LSF and considering the increasing frequency of extreme droughts, our study provides critical insights into the vulnerability and resilience of European beech in response to changing environmental conditions. Our findings demonstrate that damaging frost events in beech can be effectively reconstructed by comparing the beech tree‐ring individual series with the tree‐ring chronologies of the less frost‐sensitive Norway spruce, underscoring the value of using reference species to reveal species‐specific disturbances. By contrast, relying solely on phenological and climatic data to pinpoint damaging frost years proves challenging due to the inherent uncertainties within these predictions, as well as the difficulty in obtaining actual bud temperature, especially during nights with clear skies, where strong radiative cooling occurs.

While our tree‐ring analyses did not show a significant long‐term change in damaging LSF events at either site between 1931 and 2021, the emergence of six damaging LSF events since 1990 at the high site exceeds twice the return rate of such events over the study period. This recent increase in frost events raises significant concerns for forest ecosystems in mountainous regions. It suggests a potentially increasing frost risk, linked to climate‐induced shifts in phenology, which may undermine the resilience of high‐elevation forests under ongoing climate change, particularly when associated with the increase in the magnitude and frequency of extreme droughts.

## Competing interests

None declared.

## Author contributions

YV, EM‐S, and FB conceived the ideas and designed the methodology for this study. JR, YV, EM‐S, and FB collected tree cores in the field. JR measured tree‐ring width with the supervision of EM‐S and YV. EM‐S, LS, and YV led the data analysis. EM‐S and YV led the writing of the manuscript with substantial inputs from LS and FB.

## Disclaimer

The New Phytologist Foundation remains neutral with regard to jurisdictional claims in maps and in any institutional affiliations.

## Supporting information


**Fig. S1** Impact of the damaging spring frost that occurred on 11–12 May 2020 on the higher site at 1365 m.
**Fig. S2** Gridded vs recorded temperature.
**Fig. S3** Map of all sites with phenological observations used for model calibration.
**Fig. S4** Observed vs predicted leaf‐out dates using the M1 model.
**Fig. S5** Last annual frost events ≤ −1.0°C and predicted leaf‐out dates since 1931.
**Fig. S6** Examples of tree‐ring series from four cores of two species at two sites since 2000.
**Fig. S7** Correlations between spruce and beech chronologies in the two sites for 1931–2021.
**Fig. S8** Climate growth correlations of spruce and beech in the two sites for 1931–2021.
**Table S1** Dendroecological characteristics of the sampled trees.
Please note: Wiley is not responsible for the content or functionality of any Supporting Information supplied by the authors. Any queries (other than missing material) should be directed to the *New Phytologist* Central Office.

## Data Availability

The data that support the findings of this study are openly available at https://github.com/YVitasse/LSF‐Weissenstein.git.
