# Supplementary material for: Emerging trend of increasing spring frost damage for beech at higher elevations in the Jura Mountains: evidence from tree‐ring data
Source: New Phytol. 2025 Aug 19;248(2):600–14. doi: 10.1111/nph.70471 (PMC12445829; doi:10.1111/nph.70471)
Supplement: Supplementary file 1 — Fig. S1 Impact of the damaging spring frost that occurred on 11–12 May 2020 on the higher site at 1365 m. Fig. S2 Gridded vs recorded temperature. Fig. S3 Map of all sites with phenological observations used for model calibration. Fig. S4 Observed vs predicted leaf‐out dates using the M1 model. Fig. S5 Last annual frost events ≤ −1.0°C and predicted leaf‐out dates since 1931. Fig. S6 Examples of tree‐ring series from four cores of two species at two sites since 2000. Fig. S7 Correlations between spruce and beech chronologies in the two sites for 1931–2021. Fig. S8 Climate growth correlations of spruce and beech in the two sites for 1931–2021. Table S1 Dendroecological characteristics of the sampled trees. Please note: Wiley is not responsible for the content or functionality of any Supporting Information supplied by the authors. Any queries (other than missing material) should be directed to the New Phytologist Central Office. [file NPH-248-600-s001.pdf]

New Phytologist **Supporting Information**

Article title: Emerging trend of increasing spring frost damage for beech at higher elevations in the Jura Mountains: evidence from tree-ring data

Authors: Yann Vitasse[1,2], Lynsay Spafford[1,3], Joanna Reim[1], Frederik Baumgarten[4], Elisabet Martínez-Sancho[5]

Article acceptance date: 02 July 2025

The following Supporting Information is available for this article (short titles):

Table S1. Dendroecological characteristics of the sampled trees.
Fig. S1. Impact of the damaging spring frost that occurred on 11-12 May 2020 on the higher site at 1’365m.
Fig. S2. Gridded vs. recorded temperature.
Fig. S3. Map of all sites with phenological observations used for model calibration.
Fig. S4. Observed vs. predicted leaf–out dates using the M1 model.
Fig. S5. Last annual frost events ≤–1.0°C and predicted leaf–out dates since 1931.
Fig. S6. Examples of tree-ring series from four cores of two species at two sites since 2000.
Fig. S7. Correlations between spruce and beech chronologies in the two sites for 1931–2021.
Fig. S8. Climate-growth correlations of spruce and beech in the two sites for 1931–2021.

Table S1. Dendroecological characteristics of the sampled trees. AC, first-order autocorrelation of raw tree-ring width series; r, mean correlation of individual tree-ring width series with the mean site series.

Site	Species	Number of trees cored	Age at 1.3m in 2021 (years)	Tree-ring width (mm)	AC	r
High	Beech	12	166	0.727	0.557	0.665
High	Spruce	12	156	0.832	0.837	0.503
Low	Beech	11	118	1.190	0.704	0.627
Low	Spruce	12	115	1.345	0.779	0.563

Figure S1

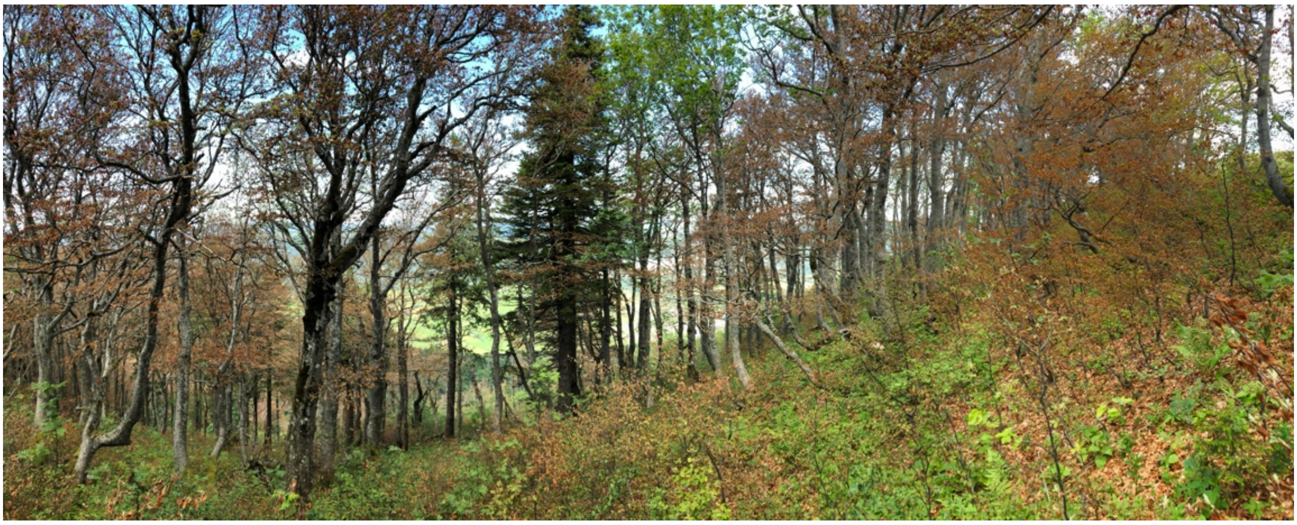

Fig. S1. Impact of the damaging spring frost that occurred on 11-12 May 2020 on the higher site at 1’365m. Picture taken on 30 June 2020. © Y. Vitasse.

Figure S2

A

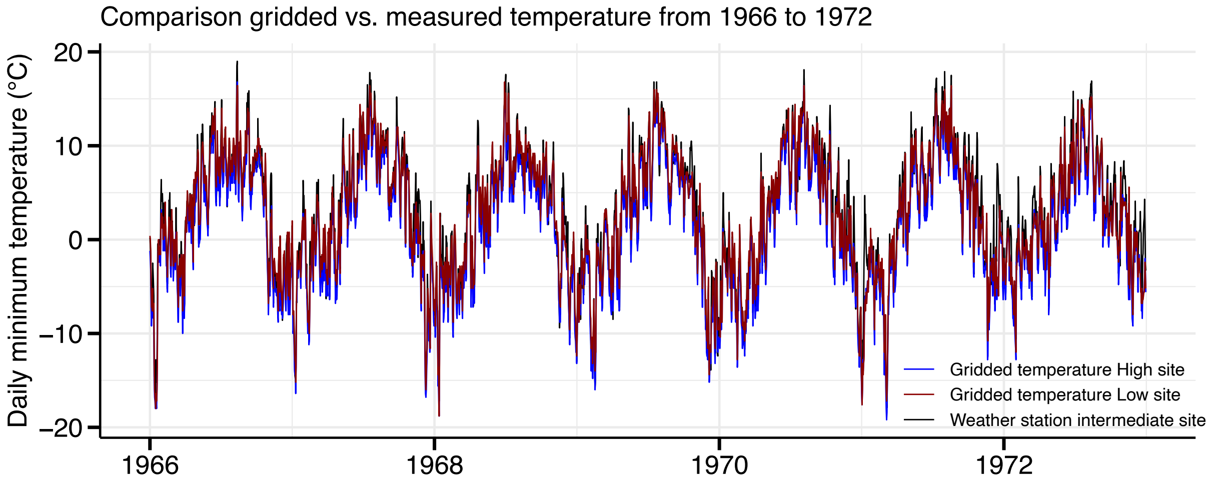

B

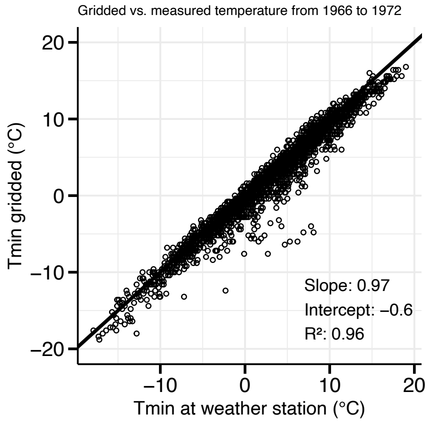

Fig. S2. Gridded and recorded temperature at the two studied site and at the historic weather station from 1966 to 1972. A, Daily temperature from 1966 to 1972 at the two studied sites (high site: 1’365m; low site:1’085m; weather station from MeteoSwiss: 1’228m). B, Correlation between daily minimum temperature recorded at the MeteoSwiss weather station from 1966 to 1972 and gridded predicted at this site from the gridded dataset.

Figure S3

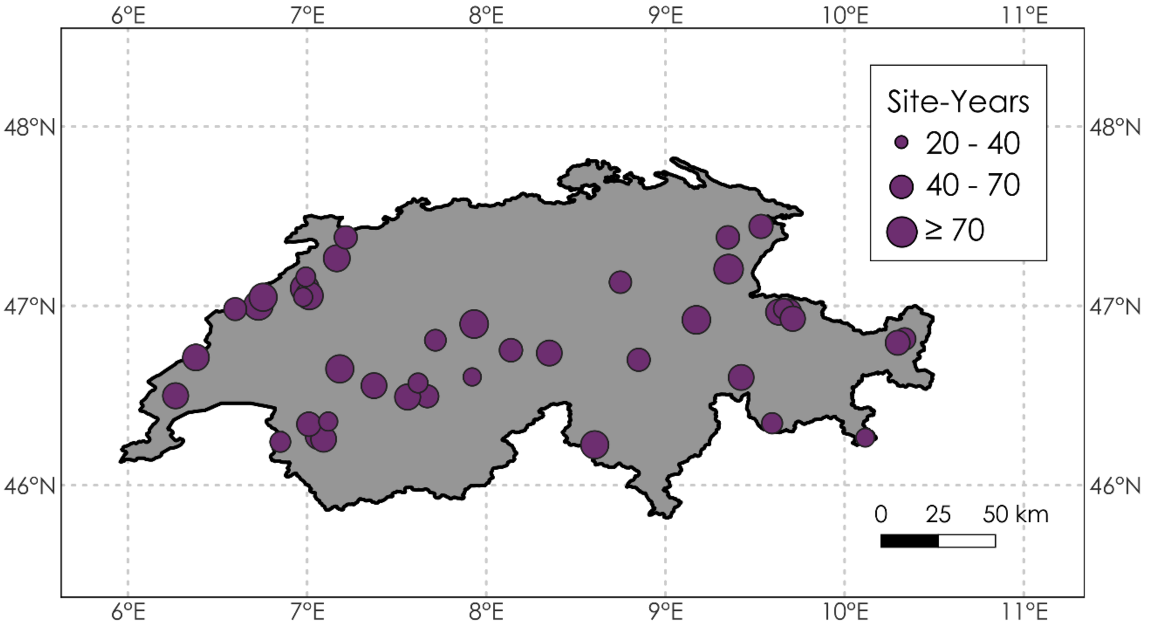

Fig. S3. The location and duration of site observations used to calibrate the M1 phenology model. Only sites above 800m ASL and having more than 20 years of observations have been selected.

Figure S4

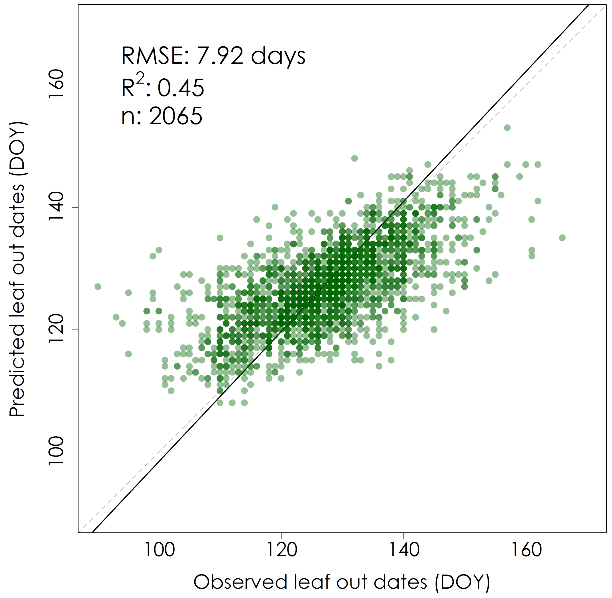

Fig. S4. Observed versus predicted leaf–out dates using the M1 model for the entire calibration dataset. The resulting optimal parameters were a start date of January 3rd, a base temperature of 0.29°C, a k parameter of 4.76, and a F_{crit} of 1248 units.

Figure S5

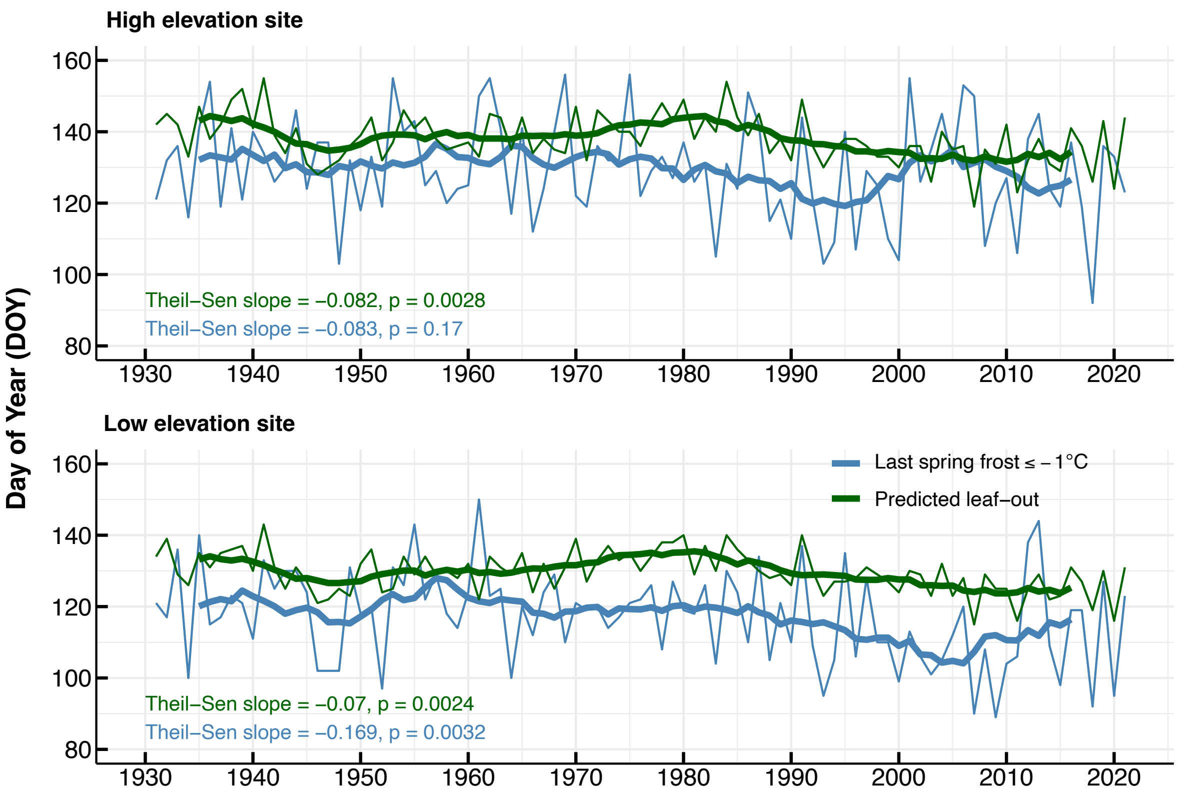

Fig. S5. Last spring frost events ≤–1.0°C (blue line) and predicted leaf–out dates (green line) for the low and high elevation site. Ten–years moving averages are represented with thicker lines

Figure S6

A. High elevation *Picea abies* #9

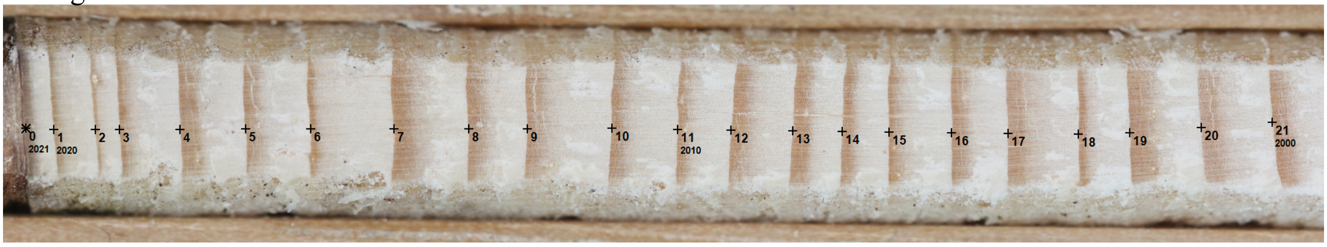

B. High elevation *Fagus sylvatica* #8

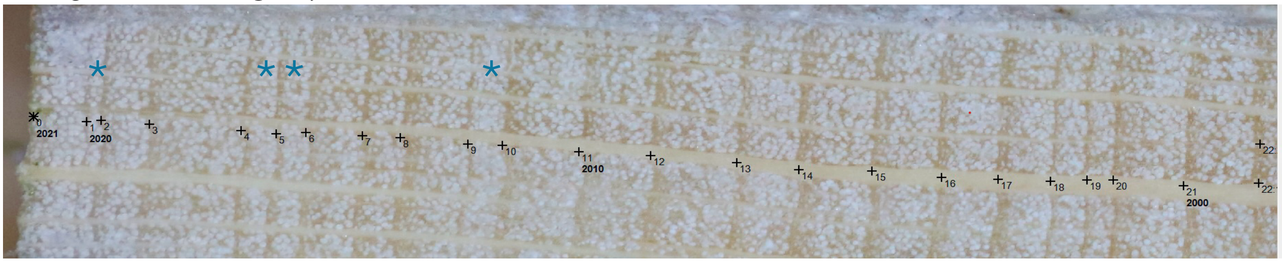

C. Low elevation *Picea abies* #6

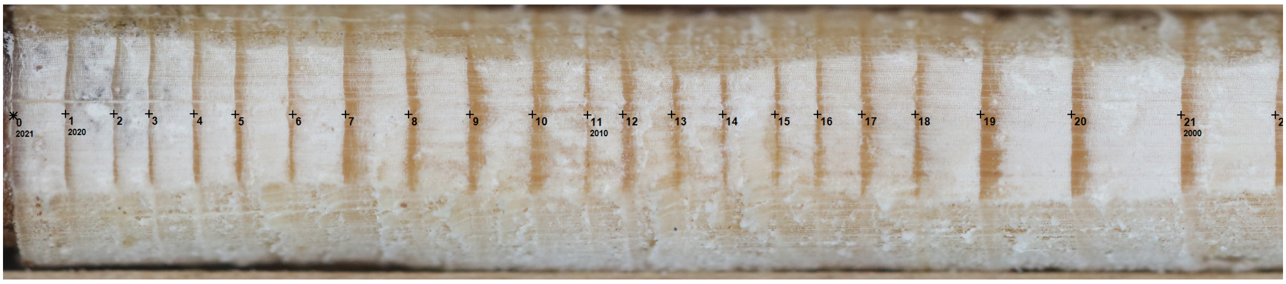

D. Low elevation *Fagus sylvatica* #5

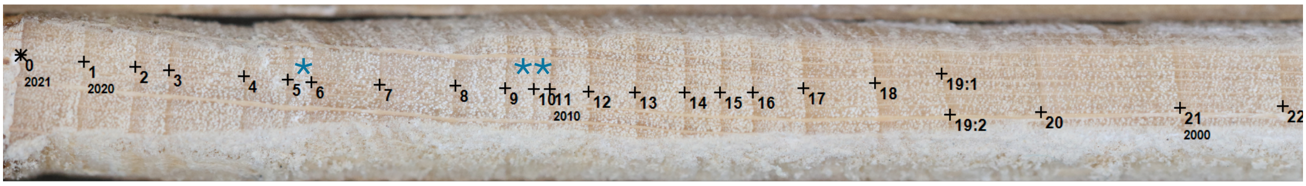

Fig. S6. Examples of tree-ring series from four cores of two species at two sites since 2000. For European beech, stars indicate frost years based on comparisons with spruce chronologies. Note that in 2020, when frost damage was visually observed by the authors only at the high site, the high site exhibits very narrow ring width, whereas the low site shows regular ring width.

Figure S7

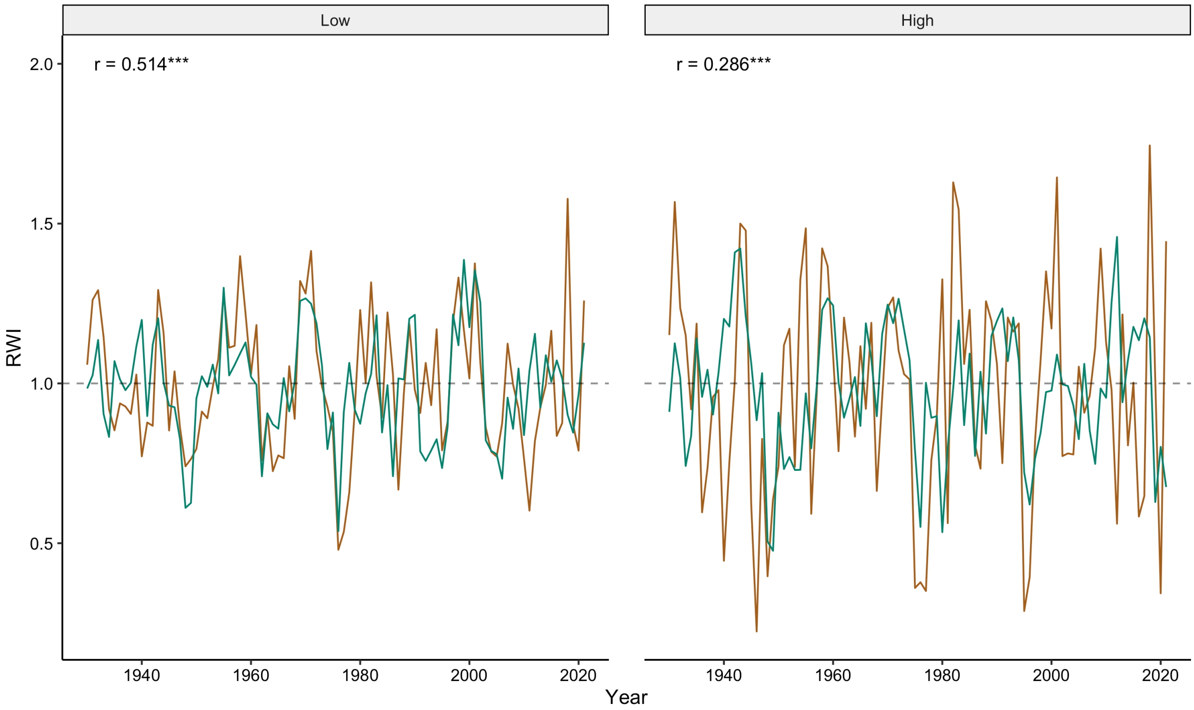

Fig. S7. Ring width index of beech (brown) and spruce (green) chronologies at the low and high site.

Figure S8

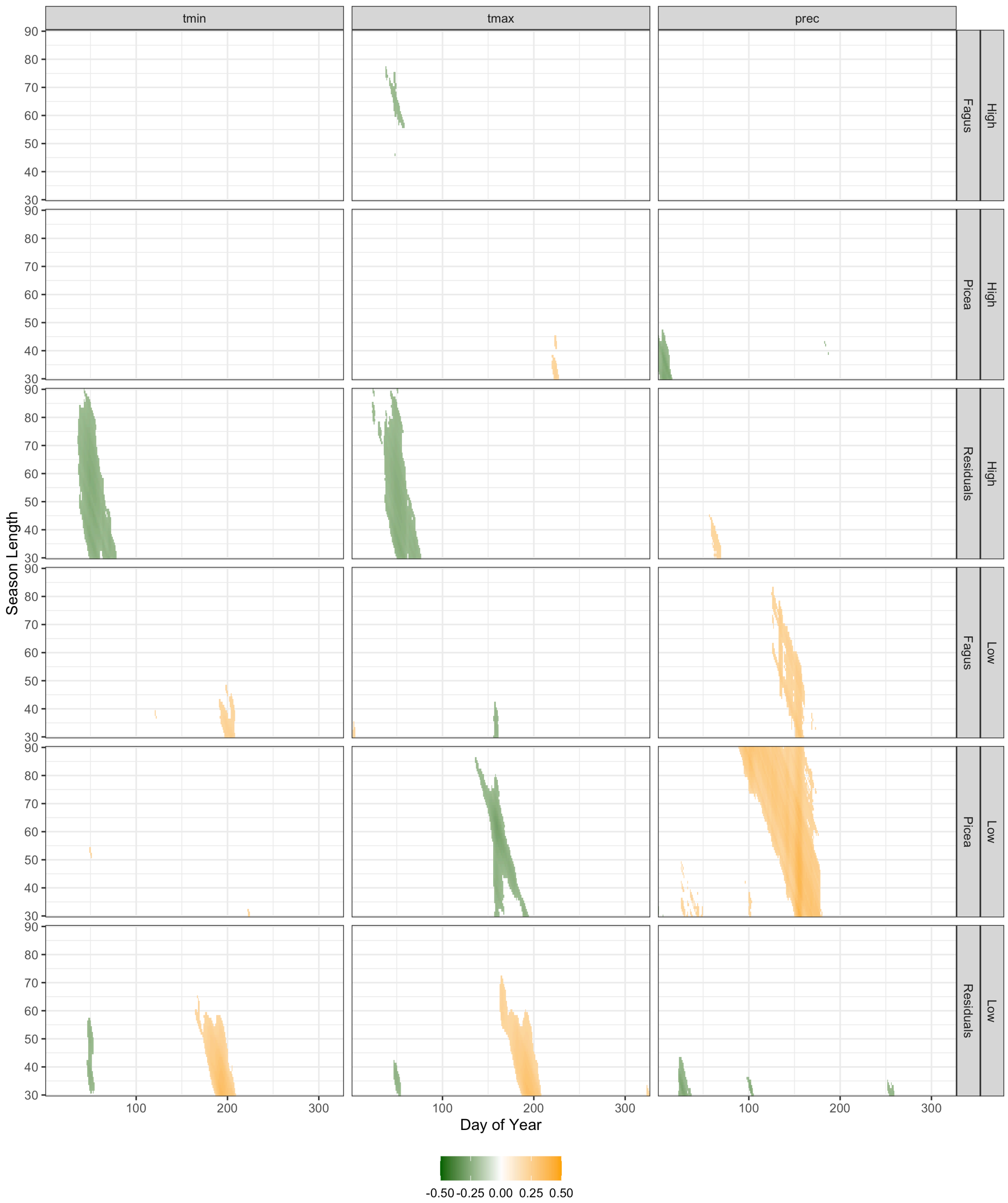

Fig. S8. Bootstrapped partial correlations between the species– and site–specific chronologies and day-wise aggregated climate data (minimum (tmin) and maximum (tmax) temperatures and precipitation) in different windows widths (season length) for the period 1931-2021. The residual chronology was calculated as the mean of all the individual beech series (RWI) after subtraction of the spruce chronology. The reference position for each value is the beginning of the considered window (season length). Only significant correlations ($p<0.05$) are shown.
